# The effects of environmental parameters on the microbial activity in peat-bog lakes

**DOI:** 10.1371/journal.pone.0224441

**Published:** 2019-10-24

**Authors:** Sylwia Lew, Katarzyna Glińska-Lewczuk, Marcin Lew

**Affiliations:** 1 University of Warmia and Mazury in Olsztyn, Department of Microbiology and Mycology, Faculty of Biology and Biotechnology, Olsztyn, Poland; 2 University of Warmia and Mazury in Olsztyn, Department of Water Resources, Climatology and Environmental Management, Faculty of Environmental Management and Agriculture, Olsztyn, Poland; 3 University of Warmia and Mazury in Poland, Faculty of Veterinary Medicine, Olsztyn, Poland; University of Shiga Prefecture, JAPAN

## Abstract

Microbiological activity is an important parameter for understanding the functioning of different environments. Therefore, the purpose of this study was to estimate the quantity and contribution of metabolically active at the single-cell level bacteria in the microbial community in peat-bog lakes. To determine different aspects of the metabolic activity of bacteria, four fluorescent staining methods (Dehydrogenase/Electron Transport System Activity -CTC+, Nucleoid Containing Cells- NuCC+, Active Bacteria with Intact Ribosome Structures- RIB+ and Active Bacteria With an Intact Membrane—MEM+) were applied. We identified four natural peat-bog lakes in Northern Europe to determine which factors—community (bacterial factors) or environment (hydrochemical and physical factors)—have a significant influence on the quantitative dynamics of metabolically active microorganisms, in terms of seasonal and habitat changes. The results show that change in the amount of abiotic components such as DOC, TN, and TOC can result in stress, which may limit a function but does not lead to losing all other metabolic functions in the community-forming bacteria. In nutrient-poor peat bog lakes, nutrients and organic carbon are factors which regulate the overall activity of the community.

## Introduction

During the last few decades, interest in the diversity, complexity and activity of microbial systems has dramatically increased, largely due to the development of new molecular techniques in environmental microbiology. Freshwater microbial populations, as the foundation of freshwater food webs as well as primary biogeochemical agents involved in nutrient cycling, have also attracted attention, but to date there has been considerably less research on these populations than marine microbial communities [1]. From amongst the diversity of freshwater ecosystems, dystrophic lakes—which have an acidic pH and are rich in humic matter—remain yet relatively understudied.

For a long time, microbiologists have pondered over the issue of bacterial activity and whether most bacteria in the environment are "active", "inactive", or "dormant" [2]. This has clear practical implications in the fields of sanitary, clinical, food and environmental microbiology, as well as having ecological implications. This issue began to be seriously discussed regarding aquatic microorganisms more or less at the time that scientists were developing new methods for estimating the abundance of water bacteria [3]. The number of ecosystem communities rarely correlates with the measurement of their activity, and since this parameter is essential for understanding the ecological role of microorganisms and their contribution to the processes which take place in the studied ecosystem, it is necessary to determine bacteria metabolic state at the single-cell level [4–5]. There are increasing numbers of techniques available to probe the activity and characteristics of single bacterioplankton cells. In environmental studies, methods such as fluorescent microscopy are widely used. Among techniques used for the analysis of an aquatic community is electron transport activity (dehydrogenase activity), wherein fluorogenic tetrazolium salt- CTC (Triphenyltetrazolium chloride), is used. CTF is a formazan dye which is the product of a chemical or biological CTC reduction. It is deposited intracellularly, and is fluorescent red in color. This technique allows us to estimate metabolic bacterial activity in aerobic and anaerobic conditions, and the attuned salt concentration of CTC enables bacteria to divide and does not result in the death of the *Protozoa* which graze on them [5–6]. The CTC method has been criticized for either the potential cellular toxicity of the CTC or the low numbers of CTC-positive cells, which were frequently recorded in field studies [7–8]. It is also claimed that the CTC method seems to focus only on the cells with the highest respiration rate [9–11]. Another way of monitoring microorganism activity is by using a biological index of membrane permeability-integrity and functionality [11]. Due to a major role the bacterial membrane plays in the functioning of the cell, its state provides information on the general physiological condition of the cell. There are a number of aspects of membrane function which are in the scope of ecological interest, e.g. membrane integrity, energization and polarity as well as factors such as the pH and ionic gradients [3]. It is assumed that bacteria are potentially active if their cell membrane is intact (MEM+), whereas they are dead if their cell membrane is damaged (MEM–) [12]. The analysis of cell membrane continuity is based on their ability to exclude various chemical compounds, such as fluorescent dyes, which normally, even in low concentrations, do not pervade undamaged and well-functioning cell membranes [5]. Fluorescent dyes also allow us to estimate the number of active cells in the community by identifying integrated DNA (NuCC). After staining, the tightly packed DNA creates a highly fluorescent structure in the cell as a result of the A-T sequence, whereby we can achieve permanent staining with the fluorochrome DAPI (4',6-diamidino-2-phenylindole). Empty cells, known as ‘ghosts’, are unable to grow, whereas nucleoid cells (NuCC) are capable of metabolic activity. Damaged, starving, or virus infected cells are usually classified as metabolically inactive. A similar effect can be seen in cells whose DNA is less tightly-packed or is insufficient in concentration [11]. Additionally, the intact structures of ribosomes may indicate the physiological state of the cell [13]. Only viable and highly active cells have a sufficient number of undamaged ribosomes (RIB+) to allow in situ hybridization (FISH) with the LSI (Locus Specific Identifier) [5,14]. In most studies where the FISH method is used, probes are targeted at rRNA, which is copious and characterized by structural and functional conservatism for all bacteria [15]. The use of rRNA measurements as an indicator of protein synthesis potential helps researchers create a base thanks to which the dynamics of the microorganism community can be understood [16]. Of course, the use of this method has opponents because there is not always a correlation between real-time activity and rRNA in trials, which is caused by different life strategies and non-growth activities [16].

A single cell in the environment undergoing physiological changes is a dominant strategy to deal with limited resources in the ecosystem and to escape from predators, viral infections, or any other negative biological interactions. Low activity and ultimately the dormant state are long-term survival and protection strategies, whereas high metabolic activity increases potential growth and susceptibility to predation and death [3]. Previous studies of the proportion of active bacteria in natural environments analyzed freshwater and seawater [17–20], and sediments or soil [21–23]; however, peat bog lakes have not been widely investigated. Estimating the fraction of microorganism that are live/dead and active/dormant may be important for our understanding of peat-bog ecosystem functioning, but microbial activity levels and population dynamics are embedded in a web of changing ecological and biological relationships [24]. Two major groups of factors regulate the physiological structure of bacterioplankton, i.e. cell distribution in the community depending on various physiological categories. The level of activity and physiological reactions of individual bacterial cells are influenced by environmental and phylogenetic factors; there is little likelihood that cells from different bacterial strains and taxa will react identically to environmental stimuli. There are many coexistent bacterial phylotypes in each bacterioplankton community; therefore, there have to be many metabolic and physiological answers for any combination of environmental factors, e.g. pH, temperature, rate of supply, and nature of the organic substrates and nutrients [3]. On the other hand, the physiological structure of bacterioplankton is regulated by factors that influence the activity that is lost intentionally, because cells of higher metabolic level are consumed by protists and infected with viruses [25]. Therefore, the contribution of these cells depends not only on cell division and the activity rate, but also on the environment and the number of losses [5].

Because microbiological activity is a crucial parameter for the functioning of aquatic ecosystems, tracing the significance of various habitat changes for the whole environment is a serious research challenge. Peat bog lakes are especially important for the areas nearby watershed divides, where they are located, due to the natural ability to increase the water retention. These rainwater-fed ecosystems, are also natural filters which permanently retain and exclude excessive amounts of various organic compounds. Organic matter stored as peat deposits and other organic sediments limits their cycle. It is widely commented in the literature, that the intensity of anthropopression, which is the effect of draining and exploiting the ecosystems, contributes to releasing significant amounts of carbon dioxide, nitric oxide, and methane; however, well-functioning, active wetland ecosystems contribute to limiting the greenhouse effect [26]. They constitute a place for the transformation of nutrients, organic compounds, metals, and organic matter components. The processes taking place in wetlands play an important role in the global cycles of carbon, nitrogen and sulphur, transforming them and releasing them into the atmosphere. Microorganisms, which are numerously represented in wetland ecosystems, are responsible for the efficiency of these processes [27].

Since microorganism activity is an essential parameter for understanding the ecological role of bacteria in aquatic ecosystems, the aim of our paper was to estimate the quantity and contribution of metabolically active at the single-cell level bacteria in the microbial community. Our research was conducted in natural peat bog lakes with no direct anthropogenic influence, in order to determine which of the community (bacteria) or environment (hydrochemical and physical) factors has a significant influence on the quantitative dynamics of metabolically active microorganisms, in terms of seasonal and habitat changes.

## Materials and methods

### Research area and sampling

The study was carried out on four natural peat-bog lakes located in the Wigry National Park, in the northeast of Poland, Europe. A permission to conduct research in the Park was issued by the Management Board of Wigierski National Park and the documentation is kept in the Park’s archives. In these lakes, dystrophication and acidification of the water are caused by natural processes resulted from ombrotrophic water supply of the relatively small drainage basin, not by the anthropogenic changes driving the acidification of many lakes in Northern Europe. The lakes are poor in nutrients but simultaneously show high humic matter content, from a peat moss floating mat surrounding the lake. Particles of humic acids suspended in lake water absorb its nutrients, and their surplus makes the water acidic and brown-stained (coca-cola color-like) and limits the light [28, 62]. Basic morphometric data and the locations of the studied lakes are shown in Table 1.

**Table 1:** Basic morphomertric parametres and geographical location of the studied lakes.

Lake name	Lake area (ha)	Max depth (m)	Geographical coordinates of lake location
Suchar II	2.50	9.5	54° 05′ 14″ N 23° 01′ 03″ E
Suchar III	0.33	3.0	54° 05′ 19″ N 23° 01′ 18″ E
Suchar IV	1.15	8.0	54° 05′ 23″ N 23° 01′ 29″ E
Wygorzele	2.00	2.5	54° 01′ 30″ N 23° 08′ 53″ E

The samples were collected seasonally (four times a year) over a three-year period. The lakes studied are shallow waterbodies which are surrounded by a peat moss zone comprised of different S*phagnum* species and shadowed by dense pine stands. The characteristic feature of the lakes was darker or brown water color as well as a well-oxygenated and warm layer, and a shallow euphotic zone. At the depth of 0.15 m the samples were collected. They were taken in the central part of each peat-bog lake from above the bottom layers by using Ruttner Water Sampler, four times during each year of the study. Also, three replicates were carried out for each single test. Samples were collected in sterile containers and immediately transported to the laboratory.

### Environmental conditions and Physico-Chemical parameters

Water temperature, pH, dissolved oxygen (DO) concentrations, and water color were recorded using a multi-parameter YSI 6600 probe (YSI Inc., Yellow Springs, USA) directly when sampling. Water color was measured with the spectrophotometer DR/2010 (HACH, Loveland, CO, USA) using the APHA platinum-cobalt standard method, the attenuated radiation method (direct reading), and the HACH sulfide test [29]. The levels of TOC (total organic carbon), DOC (dissolved organic carbon), TP (total phosphorus) and TN (total nitrogen), were measured with a spectrophotometer (Shimadzu UV–VIS 1601, Shimadzu Europa GmbH, Duisburg, Germany) and a Shimadzu TOCV-CSH total organic carbon analyzer [30]. All samples were analyzed in triplicate.

### Microbiological parameters and epifluorescence microscopy

#### Bacterial abundance (DAPI)

The bacterial abundance (DAPI) was determined using a method from Porter and Feig [31]. Triplicate sub-samples (50 mL) were fixed with neutralized formaldehyde (pH 7.4) at a final concentration of 4%. In the laboratory, the samples (1 mL) were stained with DAPI (final concentration 1 μg/mL^−1^) for 15 min in the dark and gently filtered through 0.2 μm black nucleopore filters. The bacteria were counted using an Olympus BX41 epifluorescence microscope. More than 1000 bacterial cells were counted in 20 microscope fields.

#### Nucleoid containing cells (NuCC+)

Estimating active cells with integrated nucleoids is based on the presence of a visible nucleoid region [32–33]. Peat-bog lake water samples were fixed with 10% (final concentration) buffered formalin and stained with DAPI (10 ug/ml final concentration) in the dark for 5 min, and then filtered onto a 0.2 μm black Nucleopore filter (Millipore). The microorganisms on the filter were washed five times with 1 ml hot (65–70 °C) iso-propanol. The filter was dried in the dark and mounted on a microscope slide, then observed by DAPI fluorescence under epifluorescence microscopy (Olympus BX41). Cells with yellow stained interiors were counted as NuCC positive (NuCC+), or active, cells.

#### Dehydrogenase/Electron Transport System Activity (CTC+)

The abundance of respiring bacteria was determined using the probe 5-cyano-2,3-ditotyl tetrazolium chloride (CTC, Polyscience), an indicator electron transport system of respiratory activity. CTC assays were performed using a procedure modified from Rodriguez et al. [34]. A stock solution of 50 mM CTC (Polysciences) was prepared daily, filtered through 0.1 μm, and kept in the dark at 4 °C until use. The CTC stock solution was then added to 2 mL of sample (5 mM final CTC concentration) and incubated for 4 h at the in situ temperature in the dark. The red fluorescence of the CTC assays was used to discriminate the CTC positive cells (CTC+). All bacteria were counterstained by DAPI. The percentage of CTC positive cells was calculated relative to the total bacterial counts in the stained DAPI.

#### Active Bacteria With an Intact Membrane (MEM+)

To identify bacteria with an intact membrane we used Live/Dead BagLight ^®^Bacterial Viability Kits (Molecular Probes, Inc. Oregon). The kit consists of two fluorochromes—SYTO 9 and PI (Propidium Iodide)—which differ in spectral characteristics and their capability in penetrating healthy bacterial cells [35]. Green fluorescent SYTO 9 is a nucleic acid stain, which pervades the intact walls of viable (live) cells. It stains both DNA and RNA, and it accumulates once it has pervaded cell membranes. It is visible with Narrow Blue (NB) excitation. Red fluorescent Fluo IP Propidium Iodide pervades only dead cells with damaged cell membranes [49]. If both stains are present in the cell, they compete over staining of the nucleoid acids. IP causes the reduction of SYTO 9; therefore, membrane-damaged or dead bacteria are visible as red cells under the Wide Green (WD) filter set. Unfortunately, this technique makes it impossible to detect dead cells which have lost their cytoplasmatic contents [5,35]. In accordance with the manufacturer’s product sheet, a mixture of equal volumes of SYTO 9 and PI was prepared. A 3 μL portion of staining solution was added to 1 mL of the sub-sample (final concentration SYTO 9: 5 mM, PI: 30 mM) and the sample was incubated for 15 min. The filters were embedded in BacLight mounting oil instead of immersion oil. Green fluorescing cells were counted as membrane intact (MEM+, or “viable”), while red cells were counted as membrane compromised (MEM–, or “dead”).

#### Active Bacteria with Intact Ribosome Structures (RIB+)

The number of RIB+ was investigated using fluorescent *in situ* hybridization (FISH) with the use of Cy3-labeled oligonucleotide probes, in accordance with the hybridization procedure for aquatic microorganisms proposed by Pernthaler et al. [36–37]. Samples for the number of RIB+ were fixed in freshly buffered prepared paraformaldehyde (pH 7.4) to a final concentration of 2% (vol/vol), and stored for several hours at 4 °C. The samples were filtered through white polycarbonate filters (type GTTP; Millipore), rinsed twice with sterile water, dried at room temperature, and stored at –20 °C. The rRNA-targeted oligonucleotide probes were used to determine the bacteria belonging to the Eubacteria group, using an EUB 338 _I-III_ probe [38]. Bacterial cells on the filter sections were observed with an epifluorescent microscope equipped with filter sets for DAPI and Cy3. The fractions of FISH-stained bacteria in at least 1000 DAPI-stained cells/sample were quantified.

### Statistical analyses

The relation between active bacteria and the physical and chemical properties was assessed using a correlation analysis between each chemical and physical parameter and the frequency of occurrence of metabolically active bacteria in each lake sample.

The normality data distribution was verified with the Shapiro-Wilk test (*p* < 0.05) were followed by the statistical analyses. The dataset has been transformed into log (x + 1) in order to stabilize the variance. A one-way analysis of variance (ANOVA), with the Tukey’s multiple comparison test (*p* < 0.05) as a post-hoc procedure, was applied to test differences in bacterial abundance and water quality parameters measured at the subsurface (oxic) and the near-bottom (anoxic) layers in each lake. We also performed a two-way analysis of variance (MANOVA) to test differences in the metabolically active bacteria and environmental variables between the sites and seasons. Both the analysis of variance and correlation analyses were performed with the software package Dell^™^ Statistica^™^ 13.1.

Different environmental variables, identified as influencing bacterial activity, were tested by the method of partial least squares regression (PLS-R), which works well with short data sets [39–40]. The PLS-R is a useful method to predict dependent variables (X) from a large set of independent variables (Y). The PLS-R is belongs to regression-like techniques which have been dedicated to datasets with the multi-colinearity problem or variables that are not normally distributed and deviate from normality.

A part of the PLS-R analysis is the procedure of the Variable Importance for the Projection (VIP) which estimates the importance of each explanatory variable (X) in the model. The variables with **VIP** scores >1 indicate good candidates to explain Y, and contribute significantly to the model [41].

The Variable Identification (VID) procedure [42] was also applied to select the most important explanatory components for bacteria activity in the surface and bottom layers in the lakes. VID coefficients are correlation coefficients between the original X variables and the Y variables predicted by the PLS-R model. The variable which has got a VID coefficient > 0.80 in absolute value indicates its potential relevance as a predictor variable. All PLS-R analyses were performed using the XLSTAT version 2018.3 software for data analysis provided by Microsoft Excel^®^ by Addinsoft.

## Results

### Environmental conditions

The location of the studied peat bog lakes in the temperate climate zone causes that they are subject to significant seasonal variability of thermal conditions. In winters they are usually covered with ice what prevents the mass of water from mixing. In summers, due their shallow depths, their water has a tendency to mix down to the bottom and no clear stratification appears. Except of winter, water at the surface is significantly warmer and better aerated compared to bottom. Nevertheless, the seasonal differences in mean temperature is of statistical significance at *p* <0.001.

The lake water is dark brown and the color generally changes from 173 mg Pt L^–1^ at the surface to 350 mg Pt L^–1^ near the bottom (Table 2). The lakes are naturally acidic with a pH between 4.35 and 4.55 in the whole water column. Due to a high loading of terrestrial carbon, which the lakes receive from the surrounding pine forests and *Sphagnum sp* mat, the total organic carbon concentrations varied from 20.91 ± 9.49 mg C L^–1^ to 34.87 ± 16.78 mg C L^–1^ in the surface and bottom water layers, respectively. All data are given with ± SD (Table 2). Dissolved organic carbon was on average from 83–85% of the TOC. The water of peat bogs is characterized by low mineralization and nutrient abundance, as is typical of ombrotrophic lakes characterized by advanced dystrophy. The differences in TN concentrations between the surface and bottom zones were statistically significant (*t*-test, *p* <0.001). No such statistically significant differences were found for TP, in which values remained at a relatively stable level (ca. 0.11 mg P L^–1^) along the water column regardless of season. More detailed physical and chemical properties of the studied peat bog lakes have been presented by Lew et al. [27].

**Table 2:** Physicochemical parameters (mean ± SD) of water at the surface and bottom zones of peat bog lakes. Differences between the groups were evaluated with the *t*-test (*p* <0.05) for independent samples. Denotations: EC—conductivity, DO—dissolved oxygen, DOC—dissolved organic carbon, TOP—total organic phosphorus, TON—total organic nitrogen, N—number of samples. Significant p-values (p < 0.05) are depicted in bold italics. Asterix (*) at pH denotes median value.

Parameter	Unit	N	Surface		Bottom		
			Mean	±SD	Mean	±SD	*p*
**Temp**	°C	48	12.64	7.99	7.36	3.88	***0*.*002***
**pH**	-	48	4.35*	-	4.55*	-	0.214
**DO**	mg L^-1^	48	8.85	2.78	2.92	1.99	***<0*.*001***
**TOC**	mg L^-1^	48	20.91	9.49	34.87	16.78	***<0*.*001***
**DOC**	mg L^-1^	48	17.34	8.38	29.97	14.62	***<0*.*001***
**TN**	mg L^-1^	48	0.82	0.27	2.06	1.39	***<0*.*001***
**TP**	mg L^-1^	48	0.11	0.14	0.12	0.04	0.476
**Color**	mg Pt-Co L^-1^	48	173.33	85.45	350.11	263.66	***<0*.*001***

### Microbiological parameters

**Fig 1:**
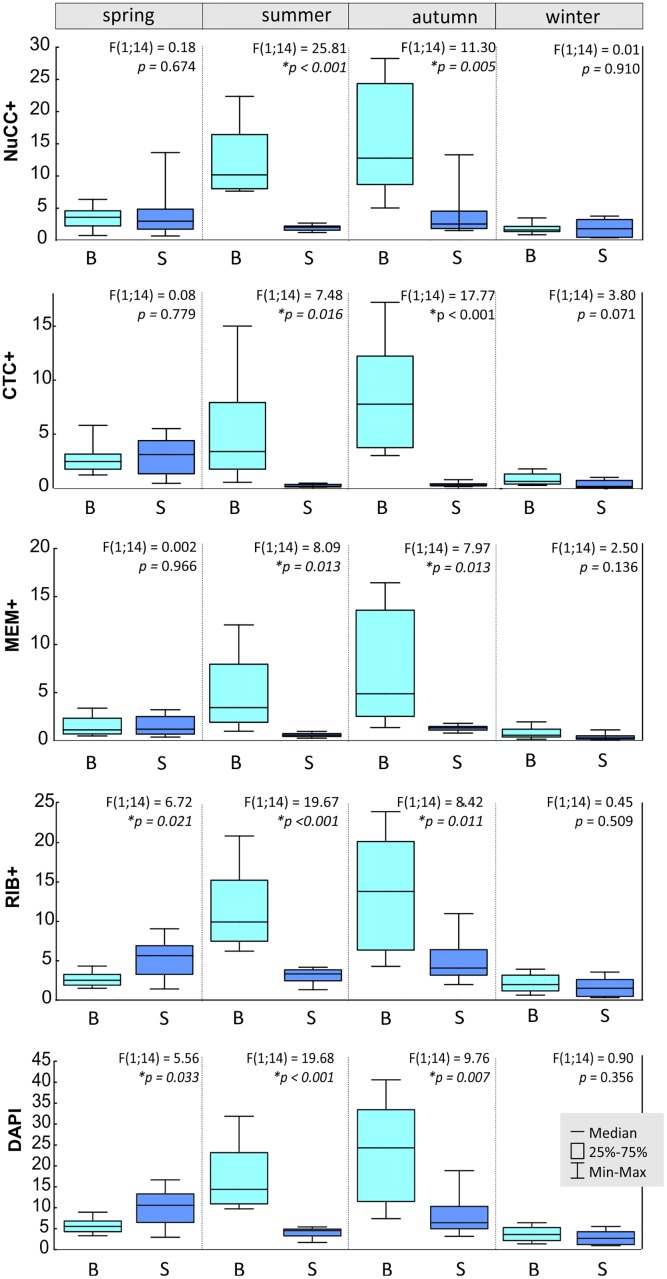
Seasonal and habitat (sites, x-axis: B- bottom, S- surface) quantitative dynamics of the total number of bacteria (DAPI in x10^6^ mL^-1^) and metabolically active bacteria (CTC+, NuCC+, RIB+,MEM+ in x10^6^ mL^-1^).

**Fig 2:**
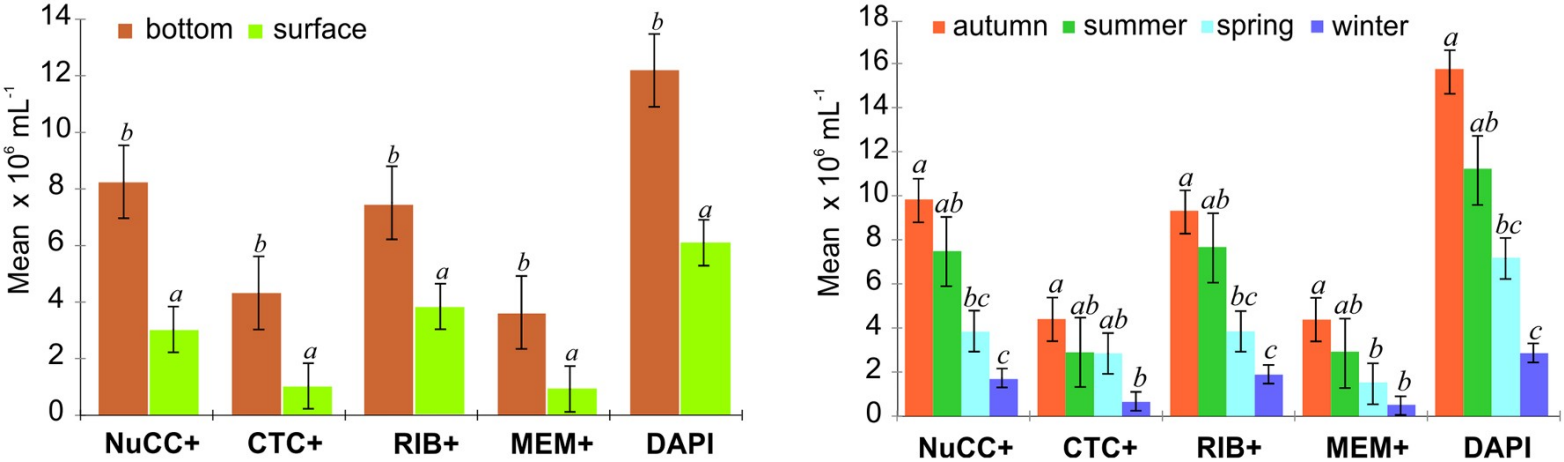
Quantitative differences between metabolically active bacteria between (a) sites and (b) seasons. Each column and vertical bar indicate a mean ± standard error.

The observations of microorganism abundance in peat bog waters showed seasonal and site diversity (Figs 1, 2a and 2b). In the surface zone, the number of bacteria was on average 6.01x10^6^ mL^-1^ (Table 3), whereas near the bottom there were almost twice that number (12.23x10^6^ mL^-1^). In the latter zone, it was observed that the highest bacteria abundance occurred in summer and autumn. In the subsurface zones of reservoirs, summer was also the season with a higher number of bacteria in the community, but the maximum abundance was in the surface zone in spring (Fig 1). The smallest habitat diversity in the number of microorganisms was observed in winter, when no significant statistical differences between the surface zone and the near bottom zone were noted (Fig 1).

**Table 3:** Comparison of DAPI, metabolically active bacteria abundance and relative abundances of bacteria communities (for DAPI, CTC+, NuCC+, RIB+,MEM+ in x10^6^ mL^-1^; other bacteria communities in %; ±SD) in water sampled from the surface and bottom layers of 4 peat bog lakes with the use of *t*-test (p<0.05).

Parameter	N	Surface		Bottom		*p*
		Mean	±SD	Mean	±SD	
**DAPI**	48	6.01	4.59	12.23	11.21	0.005
**CTC+**	48	0.98	1.47	4.31	4.65	**<0.001**
**NuCC+**	48	2.96	3.04	8.26	7.85	<0.001
**RIB+**	48	3.73	2.53	7.44	6.96	0.006
**MEM+**	48	0.93	0.75	3.66	4.57	0.001
**%CTC+ of DAPI**	48	14.00	11.80	36.46	15.93	<0.001
**%NuCC+ of DAPI**	48	48.86	18.03	65.68	14.91	<0.001
**%RIB+ of DAPI**	48	65.22	8.10	59.56	6.89	0.003
**%MEM+ of DAPI**	48	16.23	7.75	25.73	12.86	<0.001

The “physiological structure” of peat bog lakes showed the presence of numerous metabolically active bacteria in the community. Both in the surface and near the bottom, the most numerously represented are NuCC+ and RIB+ bacteria (Table 3). A statistically significant difference was observed in the proportion of these bacteria in the surface and near bottom zone of the studied reservoirs. In the upper zones of the lakes, RIB+ bacteria were most numerous (on average 3.73x10^6^ mL^-1^), which constituted over 65% of the community (Table 3). However, near the bottom, the most abundant active bacteria were NuCC+ (8.26x10^6^ mL^-1^), constituting 66% of all microorganisms that were DAPI-stained. Bacteria with intact membranes (MEM+) were the least numerous group, constituting nearly a quarter of the community, while in the surface waters they did not exceed on average 16.5% of the community (Table 3). Statistically significant quantitative diversity was shown for all activities studied between the surface and near the bottom zone (Figs 1 and 2). Regardless of the type, all metabolically active bacteria were most numerous in deeper zones of the reservoirs (Tables 3 and 4). In this site, the highest seasonal dynamics of metabolically active bacteria was also noted. As in the case of the whole community, active microorganisms were most numerously represented in summer and autumn. The same observation was made in the surface zone in spring (Fig 1). The lowest seasonal diversity characterized the activity of CTC+ and MEM+, whose numbers were higher only in autumn, and differed statistically in other seasons (Fig 2b). A linear relationship was observed between the number of bacteria (DAPI) and the number of metabolically active bacteria in the studied peat bog lakes (Fig 4). The largest regression coefficient was found for RIB+ bacteria (Fig 4). Established linear regression models for bacterial activity with respect to DAPI originating from PLS-R are outlined in Fig 4.

**Table 4:** Summary of two-way analysis of variance (ANOVA) testing the effects of site (bottom, surface) and seasons.

Parameter	NuCC+	CTC+	RIB+	MEM+	DAPI
**R^2^**	0.404	0.316	0.399	0.321	0.396
**F**	9.813	6.706	9.630	6.867	9.522
**Pr>F**	<0.0001	0.0001	<0.0001	0.0001	<0.0001
**Layer**	15.774	16.052	10.282	12.612	10.803
	0.0001	0.0001	0.002	0.001	0.002
**Season**	7.714	3.574	9.291	4.898	9.021
	0.0001	0.019	<0.0001	0.004	<0.0001

### Environmental parameters and the number of metabolically active bacteria

**Fig 3:**
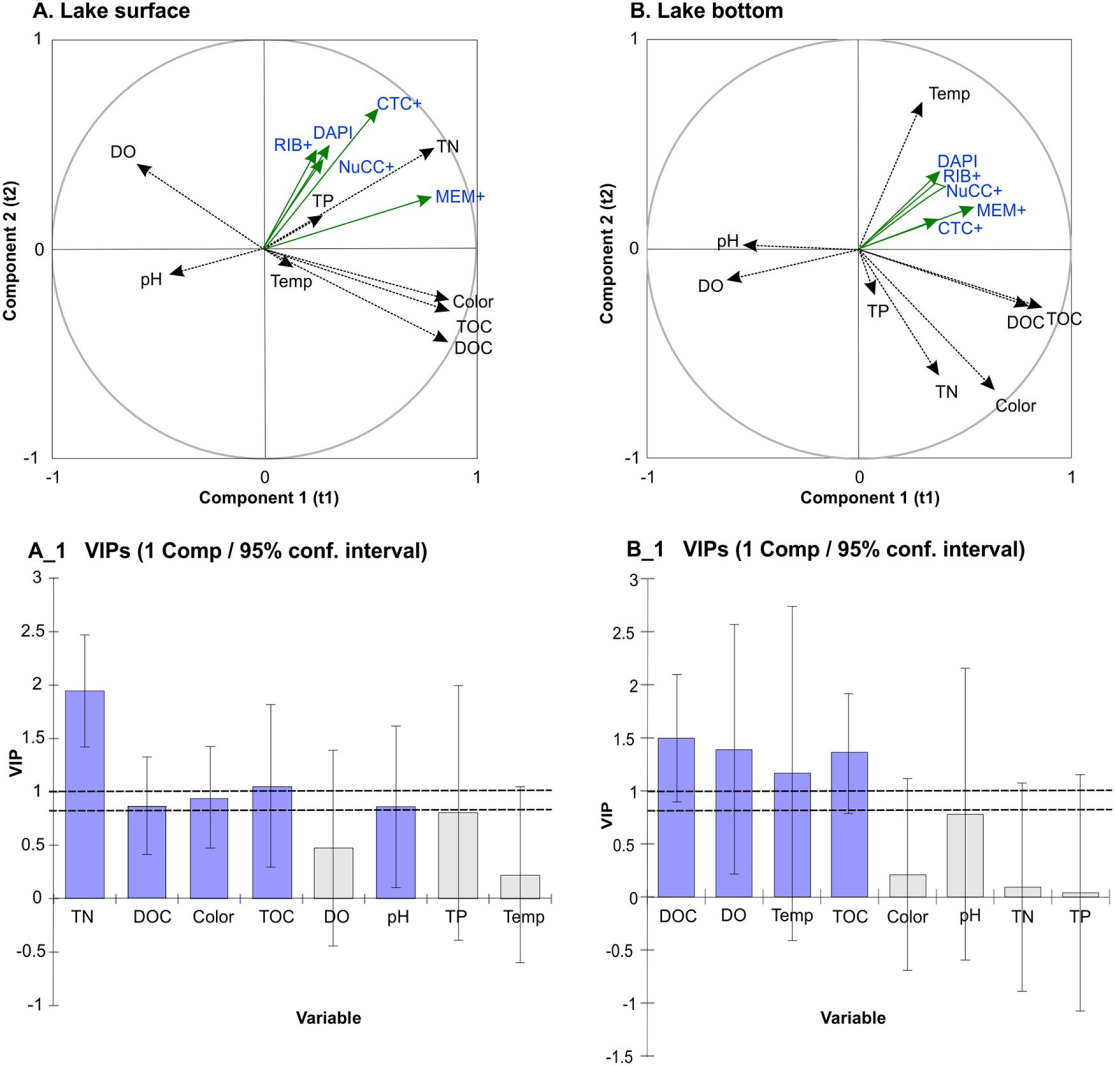
Partial least squares (PLS) regression biplots associated to the first two components: (A) Correlations with bacteria activity for surface water. (A_1) The VIPs (Variable Importance for the Projection) for explanatory variables of component 1; (B). Correlations with bacterial activity for bottom water. The VIPs (Variable Importance for the Projection) for explanatory variables of component 1 (B_1) VIPs >1 indicate the explanatory variables that contribute the most to the PLS models.

The PLS-R analysis for peat bog lakes (Table 5, Fig 3) showed that different parameters influence the community of active microorganisms in the surface zone to those in the near bottom zone. In the upper parts of the water, TN definitely influences the activity. In the subsurface zone, both the total number of microorganisms and bacteria showing different types of activity correlated positively with total nitrogen in the water (Fig 3, Table 6). This correlation was only observed in the case of total bacteria number. In both zones, carbon (TOC and DOC) was the most influential factor on the quantity contribution of groups which were the least numerous among the metabolically active bacteria (MEM+) in the community. Moreover, in the near bottom zone, influenced by the same water parameters, CTC+ bacteria came second in terms of the smallest contribution to the community. They also showed a negative correlation with dissolved oxygen DO (Table 6). RIB+ microorganisms in deeper parts of the lake were influenced by temperature. However, the dominant group among the metabolically active bacteria (NuCC+) did not depend on any parameters studied for this habitat.

**Table 5:** The percentages of the variances in X and Y explained by each variable (component 1 and component 2) in PLS-R model.

Component	R^2^ in X matrix	R^2^X(cum)	R^2^ in Y matrix	R2Y(cum)	Q^2^	Q^2^(cum)
**Lake surface**						
Comp1 (t1)	0.455	0.455	0.221	0.221	-0.246	-0.246
Comp 2(t2)	0.099	0.554	0.223	0.444	-0.558	-0.942
**Lake bottom**						
Comp 1(t1)	0.350	0.350	0.183	0.183	-0.179	-0.179
Copm2 (t2)	0.199	0.549	0.080	0.263	-0.495	-0.765

**Table 6:** Most important explanatory components for bacteria activity, selected through the VID procedure. Positive VID coefficients indicate an increase in bacteria abundance and activity, while negative coefficients denote a decrease. ±SD denotes standard deviation.

Habitat	Parameter									
	DAPI		CTC+		NuCC+		RIB+		MEM+	
	VID Y	X	VID Y	X	VID Y	X	VID Y	X	VID Y	X
**Lake surface**	0.839	TN	0.888	TN	0.847	TN	0.813	TN	0.928	TN
									0.860	TOC
									0.809	DOC
	Goodness of fit statistics R^2^									
	0.344		0.678		0.228		0.289		0.683	
**Lake bottom**	0.839	TN	0.814	DOC			0.825	Temp	0.827	TOC
			0.811	TOC					0.822	DOC
			-0.802	DO						
	Goodness of fit statistics R^2^									
	0.344		0.174		0.271		0.288		0.341	

## Discussion

Understanding environmental problems requires discussing both individual and global changes in the structure and function of land and water ecosystems [43]. Intensive anthropopression influences changes in global cycles of C, N and P, which in turn can modify chemical conditions and nutrient balance [43]. It is difficult to anticipate the consequences of these changes on the basic productivity and food web of inland waters [44]. However, to forecast more precisely the influence of these changes, it is important for us to reflect on the basic functioning of ecosystems which are crucial for the chemical element cycles in nature. Peat bog ecosystems constitute a link between water and land environments. Their biogeographical range reaches central Europe and they have important global environmental functions, such as the retention of ground and surface waters and in water purification by absorbing nitrogen and phosphorus compounds. Their tremendous importance can also be seen in climate regulation, since carbon assimilated by plants is trapped in peat deposits, which decreases the amount of atmospheric carbon. Fens are rich in different types of ecosystems characteristic of a temperate zone. They are home to numerous rare and endangered species, many of which can be found in central Europe. The importance of bacterial activity for the matter and energy cycle lies in it being the basis of almost all aquatic ecosystems [33]. It is therefore important to research the activity level of peat bog lakes and the environmental factors which regulate it, not only to understand an individual ecosystem, but to increase our understanding of global changes in the environment in order to protect it.

Many previous research studies in both marine and freshwater environments, using different staining techniques, showed a small fraction of “metabolically active” compared to total bacteria [5, 20, 33, 45]. It remains unknown the proportion of a peat bog lake community that are active bacteria. In this study, four staining methods (CTC+, NuCC+, RIB+, and MEM+) were used to determine different aspects of the metabolic activity of bacteria in dystrophic lakes, which are relatively understudied. Furthermore, water-peat bog ecosystems with small dystrophic lakes are important for their impact on climate parameters. It is connected with processes which include wetlands in global cycles of chemical elements. Organic matter stored as peat deposits and other organic sediments exclude the cycle of carbon, nitrogen and sulfur. The natural flow of carbon, which takes place where peat bogs are located, is connected with the flow of carbon in the atmosphere and methane emission, whose greatest sources are wetlands [27,62]. The reason for that is a global increase of the greenhouse effect, which poses a serious problem to the protection of natural environment. Peat bogs can serve as natural reservoirs capable of binding methane and carbon dioxide (CO2). Due to a continuous increase of greenhouse gases as a result of human activity [62], the research of these ecosystems seems to be not only justified, but also necessary.

The proportion of bacteria showing respiratory chain activity (CTC+) analyzed with an epifluorescence microscopic technique ranges from 1–50%, with an average of a dozen percent or so [5]. We obtained similar findings while researching peat bog lakes; moreover, the number of respiratory active bacteria was higher in deeper zones of the reservoirs. In freshwater studies slightly higher values, reaching up to 80% of the community, were noted in sediments [14, 22]. Observation of seasonal and habitat dynamics showed that near bottom conditions in summer and autumn are the best for CTC+ in the peat bog lake community. It seems that no elimination of active bacteria occurs in the absence of the pressure of organisms grazing on them [33, 46, 47]. Near bottom conditions might be averse to this activity. Alternatively, a positive correlation along with TBN, which we also showed in this study, might be responsible for the observed outcome. Similar to other authors, we observed that in peat bog lakes the abundance and proportion of active cells (mostly CTC+) increased with increasing lake productivity during the summer period [46, 45]. It was shown that not only the abundance of the community, but also the substrate availability, can influence the number of respiratory active bacteria [21,48].

Nucleoid-integrated cells (NuCC+), determined as metabolically active, constituted the most numerous group of active bacteria in the near bottom zone, and the second most numerous in the surface zone. The studies of freshwater and sediments showed high levels as well, reaching up to 92% in relation to the TBN DAPI-stained [32]; however, most research has shown maximum values of 43% [49] to 65% [5, 33] in lake communities with different trophic levels. It was observed that the bacteria number increases with the amount of nutrients and correlates with the trophy of reservoirs. In our study, the near bottom zone is a richer habitat in comparison to the surface zone; therefore, in the former we noted a 66% contribution of NuCC+ bacteria in the community. Bacteria which are usually determined as metabolically inactive, known also as “ghosts”, are cells which are damaged, starving or infected with viruses [5]. They were observed in peat bog lakes in temperate zones, especially in summer.

An intact ribosome structure (RIB+) in bacteria can be an indicator of the cell’s physiological state [13]. According to Blazewicz et al. [16], the number of ribosomes present at a given time limits the maximum protein synthesis activity for a population, but does not directly inform realized protein synthesis activity. Therefore, the data on the contribution of RIB+ bacteria in the community should be interpreted not as an actual activity, but as a potential one. According to these authors, if data on the number of RIB+ microorganisms are presented in comparison with the contribution of other metabolically active bacteria, they can be used as an indicator of the dynamics for organisms which propel the functions of the studied ecosystem. The most recent protocols used to identify bacteria with in situ FISH hybridization, using the EUBmix_I-III_ probe, were employed in different ecosystems to determine viable, highly active cells with the number of undamaged ribosomes needed for hybridization [11,14]. Data on soil and sediments show an 80% detection of RIB+ bacteria, but the number is lower in water. Our research shows substantial RIB+ activity, greater than in freshwater, with no quantitative differentiation between sites in spring and winter. The most favorable conditions for the activity of microorganisms were in summer and autumn, regardless of the method used. During these seasons, the least numerous group of active membrane-intact bacteria—MEM+, categorized as live cells—showed the highest values; however, only those sampled from the near bottom zone showed similar results to those obtained by other authors. Our data coincide with the level of 26–30% contribution of live cells fractions obtained by Luna et al. [50].

**Fig 4:**
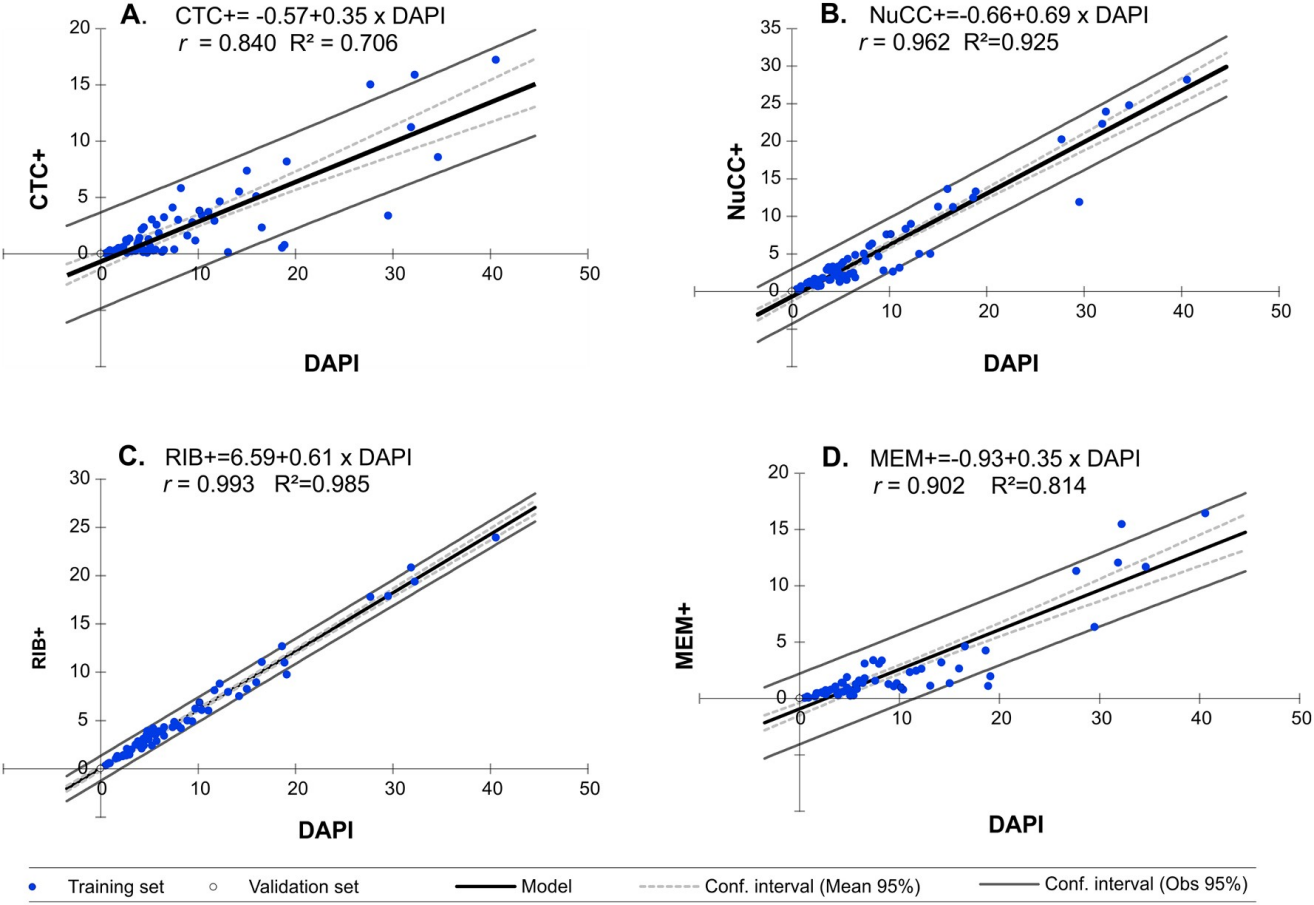
Linear regressions between the number of metabolically active bacteria CTC+, NuCC+, RIB+, MEM+ (x10^6^ mL^-1^) and the total number of bacteria (DAPI).

The obtained results show a significant positive correlation between TBN, fluorochrome DAPI-stained, and the number of metabolically active cells (Fig 4). Schumann et al. [51] studied the viability of bacteria from different aquatic habitats, and noticed that bacterial abundance and the numbers of intact and active cells increased significantly with bacterial activity [52]. In the surface zones of peat bog lakes, the number of microorganisms is correlated with total nitrogen (TN). The same correlation was valid for all types of activity. Lower total nitrogen content was observed in the surface layer than in the bottom zone. The metabolism of prokaryotes depends on the uptake of chemical compounds from the environment among which nitrogen, besides carbon, is extremely important. It is a part of cell-building compounds but it is also found in a reduced form in proteins, purines and pyrimidines that form nucleotides (DNA and RNA) or the amino sugars of cell walls. Therefore, its limited availability in the surface layer may be a factor affecting prokaryote metabolism defined by the level of an indicator of certain activity functions, such as respiratory CTC +, or the integrity of basic MEM + cell structures [6].

Only membrane-intact bacteria (MEM+) also correlated with carbon content (TOC and DOC). Such a strong correlation between bacterial activity and the production of organic matter concentrations was already noted in freshwater [51]. It was observed that all parameters of microbial activity increased significantly with POC, DOC, and bacterial abundance. This suggests that samples with high concentrations of organic matter and bacteria had not only more bacteria but specifically more active bacteria [52]. As has been generally assumed, in the method based on the integrity of cell coatings (MEM +) in both Gram + and Gram-cells, the division divides the microorganisms in the community into living and dead. In addition to the requirements associated with the construction of the membrane, which in bacteria is complicated due to the complex membrane structure [6], there is a whole series of intermediate states [63], which in the natural environment largely depend on the availability of carbon, including special forms readily available for prokaryotes. This was demonstrated in our studies where the activity determined by the MEM + method was regulated by the availability of TOC and DOC in both examined zones.

It has been known for a long time that, in aquatic ecosystems, the availability of energy—mainly in the form of organic carbon or as certain inorganic compounds or light for some bacteria—is perhaps one of the key factors influencing bacterial singe-cell activity [3]. This was proven by laboratory experiments which checked the response of cultured bacteria to extreme carbon limitation or inorganic nutrient additions [3, 53]. Our observations are the first findings on the correlation between water quality and the contribution of active bacteria in the very specific environment of nutrient-poor peat bog lakes. We showed that the bacteria activity increases in the surface zone in spring and autumn, which may result from the afflux of organic matter during the spring thaw or from dying plankton [45,54]. In this zone, nitrogen was the dominant factor in regulating metabolic activity at the single-cell level. Previous research points to the important role of nitrogen and phosphorus, which—even in minimal quantities—may regulate the growth of bacterioplankton [55–56]. This pertains to oceans that are perceived as extremely dilute and oligotrophic ecosystems. Choi et al. [57] showed, however, that a minimal amount of nitrogen and phosphorus, even without organic substances, significantly influences the growth of active bacteria. In the euphotic zone of eutrophic lakes rich in nutrients, bacterial abundance and metabolic activity were significantly controlled by the concentration of TP and DOC [46]. In humic lakes, inorganic nutrients are more important than organic substrates in regulating the growth of bacterioplankton [58] and, as our research shows, they determine bactrioplankton activity. Thus, the most important component of the studied lakes is the one with low values. In our case, it was a basic biogene—nitrogen—which regulated the growth of the microorganism population in every ecosystem [55]. Organic carbon of an autochthonous origin is of less importance, especially in the surface zone. In the narrow euphotic zone of peat bog lakes, phytoplankton and zooplankton play the most important role in providing bacterioplankton with organic substrates. DOC, which comes from secretions and excretions of zooplankton as well as cell lysis of phytoplankton [59], is sufficient to meet the bacterial demands of organic substrates, and is not the main factor regulating the metabolic activity of bacteria. Moreover, bacteria cells in this zone need to handle intermittent supplies of organic substrates and nutrients. Some of these fluctuations may exceed the protective mechanisms of the cells, with deleterious or lethal consequences to the microorganisms [60], thereby influencing both the quantitative contribution of metabolically active bacteria and their quantity in the community. The contribution of smaller bacteria in the upper zones of the reservoirs is also the effect of top-down controls. The grazing pressure of bacterivorous protists is significant in regulating the number of active bacteria, since—as was proven by del Giorgio et al. [25]—the consumption rate of metabolically active bacteria is four times higher than the consumption rate of dead or inactive bacteria. It was also proven by experiments that protozoa eliminate metabolically active cells selectively [61].

In deeper zones, due to limited light penetration and less suitable thermal and oxygen conditions for the growth of plankton, top-down control does not work; however, the organic matter from the remains of phytoplankton and zooplankton may be important for the growth of bacterioplankton. In our case, DOC and TOC regulated the chosen types of activity.

Bacteria active groups, the least numerous in the MEM+ and CTC+ community, were regulated by TN in the surface zone similarly to other active groups. In the near bottom zone, they were regulated by the availability of different carbon types. In the case of CTC + respiratory activity, starvation occurred as an important factor affecting a CTC-formazan detection level, as previously reported [14]. Fluctuations in the availability of an absorbable form of carbon is a factor that reflects the substrate becoming limited, therefore, it regulates the metabolic activity in the bottom zone.

In the eutrophic reservoir, top-down control (by flagellates and ciliates together) as well as”inside control” mechanisms (by viruses) were important in controlling bacterial community structure only in the hypolimnion, in contrast to the epilimnion where bacteria were dependent mainly on bottom-up factors [64]. In our study, due to the reservoir characteristics and thus smaller contribution of zooplankton, top-down control is not possible; therefore, the near bottom zone was rich in active bacteria. They were mostly dominant in autumn; perhaps dying phytoplankton and zooplankton in the surface zone contributed to the presence of easily degradable organic matter (labile carbon, or DOC) in deeper parts of the lakes. The growth of microorganisms and bacteria from the dormant and starvation state were stimulated. Yet another factor which regulated the number and proportion of metabolically active bacteria (RIB+) in the near bottom zone was temperature. This factor, just as with viruses, nutrients, organic substrates, and grazing by flagellates and ciliates, cannot be fully explained, although the influence of these factors on the proportion of active bacteria in bacterioplankton in trophically diversified lakes was already confirmed in short-term experiments [5, 45–46].

## Conclusion

A change in the availability of abiotic components (DOC, TN, and TOC) can result in stress, which may limit a function, but does not lead to the loss of all other metabolic functions in community forming bacteria. In nutrient-poor peat bog lakes, just as in nutrient-rich eutrophic lakes, inorganic nutrients and organic carbon are factors which regulate the activity of the community. However, in these environments the most important factor is a chemical element in shortage. In the surface zone, nitrogen definitely stimulates bacterioplankton, which makes the proportion of active bacteria in the community higher. In the near bottom zone, it is impossible to point to one factor regulating bacterial activity, although in many cases carbon activated a greater contribution of bacteria activity in the community.

## Supporting information

S1 Data Supplementary data. (XLSX)
